# The effects of tourniquet use on blood loss and perioperative complications in total knee arthroplasty

**DOI:** 10.1186/s12891-023-06979-6

**Published:** 2023-10-27

**Authors:** Yang Tan, Shun Guo, Hua Wang, Kai Tie, Jun Qin, Xinyu Zhao, Liaobin Chen

**Affiliations:** Division of Joint surgery and Sports Medicine, Department of Orthopedic Surgery, Zhongnan Hospital of Wuhan University, Wuhan, 430071 China

**Keywords:** Tourniquet, Total knee arthroplasty, Blood loss, D-dimer, Complications

## Abstract

**Background:**

There has been ongoing debate about the use of tourniquets in total knee arthroplasty, and their application is widely studied. A comprehensive understanding of the advantages and disadvantages of tourniquet use during the procedure is crucial for optimizing surgical outcomes. This study aimed to investigate the effectiveness of tourniquet application, with a particular focus on blood loss and perioperative complications, providing valuable insights for clinical practice.

**Methods:**

Fifty patients who underwent total knee arthroplasty were randomized into tourniquet (n = 25) and nontourniquet (n = 25) groups. The same surgeon performed all surgical procedures. The follow-up time was 14 days after surgery. Primary outcomes were hemoglobin level changes, blood loss, operation time, and perioperative plasma D-dimer levels. Secondary outcomes were postoperative complications, including thrombotic and nonthrombotic events.

**Results:**

No significant differences were found in drainage, calculated blood loss, total blood loss, postoperative hemoglobin levels, or blood transfusion between the two groups (P > 0.05). No differences in D-dimer levels were observed on postoperative Days 1, 3, and 14 between the two groups, except on postoperative Day 7, when the D-dimer level in the tourniquet group was lower than that in the nontourniquet group (P = 0.03). The incidence of local complications (thigh bruising, blisters, pain, fat liquefaction, and superficial infections) in the tourniquet group was significantly higher than that in the nontourniquet group (P = 0.03), but no significant differences were found in thromboembolic and nonthromboembolic events or overall complications (P > 0.05).

**Conclusion:**

We conclude that tourniquet use does not reduce the length of surgery or blood loss but does increase local complications in total knee arthroplasty.

## Background

Total knee arthroplasty (TKA) is an effective treatment to reduce pain and restore joint mobility and function in end-stage osteoarthritis (OA) [1]. Tourniquets have been used for lower limb surgery for more than a century and are still the preferred method for TKA by many surgeons [2]. Indeed, one report shows that 95% of members of the American Association of Hip and Knee Surgeons use tourniquets in TKA [3].

Tourniquet use in TKA can provide a bloodless intraoperative field and dry bone surface for optimal cement work reduce intraoperative blood loss [4]. The disadvantages of tourniquet use include complications such as skin injury (skin abrasions and blisters) (15%), nerve palsy (0.6%), deep-vein thrombosis (DVT) (3%), postoperative pain, wound healing disorders (15%), and infections (1%) [5–7]. Moreover, disagreement remains on whether tourniquet use reduces blood loss in TKA [8–10].

The pros and cons of tourniquet use are uncertain, though many studies have reported its use in TKA. As a prospective study, the primary aim of this randomized trial was to assess the efficacy of tourniquet use for blood loss in primary TKA. The secondary aim was to compare perioperative complications with or without tourniquet use in TKA. We hypothesized that nontourniquet use does not increase blood loss but does reduce perioperative complications in TKA.

## Methods

### Patient data

A prospective study involving patients with osteoarthritis who underwent primary unilateral TKA was conducted from October 2021 to December 2022 in Zhongnan Hospital of Wuhan University. Patients with cardiovascular disease, symptomatic peripheral vascular disease, oral anticoagulation use, severe knee joint deformity, and overweight (body mass index, BMI > 40) were excluded. All patients who met the study criteria were admitted to the hospital three days before surgery and discharged 14 days after surgery. Institutional Ethics approval was given by the institutional review board (IRB 2,019,018), and informed consent was obtained from each patient or candidate. The patients were randomly divided into the tourniquet or nontourniquet group in accordance with a random number table. The result of this randomization was 25 patients in each group. The patient demographics and preoperative hemoglobin levels, which matched well, are summarized in Table 1.

**Table 1:** Patient demographics for tourniquet and non-tourniquet use

	Tourniquet	Non-tourniquet	*P* value
Case	25	25	
Gender (male/female)	7/18	6/19	0.75 (Chi-square) Test)
Age (years) (IQR)	70 (62 ~ 77.5)	72 (63 ~ 78)	0.65 (u-test)
Height (cm) (IQR)	163 (157 ~ 166)	163 (160 ~ 167)	0.48 (u-test)
Weight (kg) (IQR)	69 (62.5 ~ 73)	70 (65 ~ 75)	0.49 (u-test)
BMI (kg/m²) (IQR)	25.5 (24.3 ~ 27.2)	26.0 (25.4 ~ 27.4)	0.38 (u-test)
Preoperative hemoglobin level (g/L) (mean ± SD)	123.38 ± 17.52	124.32 ± 13.18	0.83 (t-test)

IQR, interquartile range; cm, centimeter; kg, kilogram; g/L, gram/litre; BMI, body mass index; SD, standard deviation

### Surgical procedure

All patients were administered general anesthesia. All surgical procedures were performed by the same senior knee surgeon (Dr. LB C) who is experienced in the instrumentation and use of the cement PS prosthesis (Genesis II, Smith & Nephew Inc., Memphis, TN, USA). The inflation pressure of the tourniquet was 300 mm Hg. The procedure was performed via a midline skin incision and a medial parapatellar approach to the knee. The cruciate ligaments were excised. Intramedullary and extramedullary alignments were used for the femur and tibia, respectively. Appropriate soft-tissue procedures to realign the knee were performed in both groups. The patella was not resurfaced in all cases but was reshaped to better match the femoral component trochlea if necessary. Denervation was performed by using electrocautery of the patellar rim.

### Perioperative management

Color Doppler ultrasound examination was performed by experienced lower limb vascular specialists in the ultrasonography Department in Zhongnan Hospital of Wuhan University at 2–3 days before surgery to exclude DVT. Antibiotic treatment with first/second-generation cephalosporin was intravenously administered (one dose preoperatively and for the next 3 days) once before surgery (cefazolin, 0.5 g) and once every 3 days after surgery (cefazolin, 0.5 g/day). The quantity of intravenous fluids applied within 24 h postoperatively was 2500–3000 ml. Hemoglobin levels and D-dimer values were recorded on postoperative Days 1, 3, 7 and 14. Allogeneic blood transfusion was performed if the hemoglobin level was lower than 70 g/L. All patients received standard thromboprophylaxis by administration of low-molecular weight heparin. Active isometric quadriceps, initiative straight leg raising, and extending–flexing motion were encouraged. The patients were allowed to walk 3 days after the surgery with walking aids. Sutures were removed 14 days after surgery. Color Doppler ultrasound examination was performed again on postoperative Day 14 to assess the presence of DVT.

### Blood loss measurements and calculations

The calculated total blood loss (CBL) was obtained by using the Gross method [10]. Total blood loss (TBL) was the sum of the CBL and volume of blood transfused.

### Statistics of nonthrombotic complications

In this study, we defined nonthrombotic complications as local complications, gastrointestinal complications, anterior knee pain, and urinary tract infection. The surgeon determined these nonthrombotic complications through the patient’s chief complaint, signs, and test results (such as urine routine examination) during the ward round.

### Power analysis and Statistical analysis

Power analysis was performed by using GPower3.1.9.2. for outcomes of thromboembolic eventrate, overall complication rate, and local complication rate. Measurement data with a continuous normal distribution are expressed as the mean and standard deviation (SD), and Student’s t test was used. Continuous nonnormally distributed continuous measurement data are expressed as IQRs using the Mann‒Whitney U test. Count data were compared using the chi-square test. Repeated measures analysis of variance (ANOVA) was used to compare differences in hemoglobin and D-dimer levels. Data analysis was performed by using the SPSS statistical package (version 17.0), and P < 0.05 was considered statistically significant.

## Results

### Sample size

Sample calculations of the thromboembolic event rate, overall complication rate, and local complication rate were performed, namely, power analysis. The effect size was 0.881, 0.520, and 0.797, respectively. The calculated power values were 0.999, 0.957, and 0.999, respectively. The sample size calculated according to a statistical power of 0.80 required 11 cases, 30 cases, and 13 cases, respectively. Hence, our sample size (50) was sufficient to obtain results of our main observations.

### Blood loss, blood transfusion rates, and operation time

Blood loss data for the tourniquet and nontourniquet groups are summarized in Table 2. Mean postoperative hemoglobin levels did not differ significantly between the tourniquet and nontourniquet groups on postoperative Days 1, 3, 7, and 14 (P > 0.05). Drainage, calculated blood loss, and total blood loss also did not differ significantly between the two groups (P > 0.05). No differences between the two groups were found in the blood transfusion volume and rate (P > 0.05) or in the operation time (P > 0.05).

### Perioperative D-dimer levels

The perioperative D-dimer levels measured are presented in Table 3, with no differences between the tourniquet and nontourniquet groups. However, postoperative plasma D-dimer levels increased significantly and peaked on postoperative Days 1 and 7. The D-dimer level in the tourniquet group was lower than that in the nontourniquet group on postoperative Day 7 (P = 0.03), with no difference between the two groups on the remaining postoperative days (P > 0.05).

**Table 2:** Blood loss and other parameters for patients who underwent TKA with or without tourniquet use

	Tourniquet	Non-tourniquet	*P* value
**Hemoglobin level changes (g/L)**			
Postoperative day 1	95.58 ± 12.49	91.26 ± 13.33	0.24 (ANOVA)
Postoperative day 3	83.68 ± 13.63	83.06 ± 12.44	0.86 (ANOVA)
Postoperative day 7	89.01 ± 11.39	86.36 ± 12.21	0.43 (ANOVA)
Postoperative day 14	97.29 ± 8.91	93.05 ± 11.08	0.14 (ANOVA)
**Drainage(ml)**	387.08 ± 247.75	345.22 ± 138.49	0.48 (t-test)
**Calculated blood loss (ml)**	1369.02 ± 563.72	1451.44 ± 347.80	0.54 (t-test)
**Total blood loss (ml)**	1529.02 ± 735.37	1599.44 ± 450.27	0.69 (t-test)
**Blood transfusion rate**	8/25	7/25	0.76 (Chi-square test)
**Blood transfusion(ml)**	550.00 ± 434.25	528.57 ± 170.43	0.90 (t-test)
**Operation time (min)**	99.08 ± 13.72	92.28 ± 10.76	0.06 (t-test)

ANOVA, analysis of variance; g/L, gram/litre; ml, milliliter; min, minute. Measurement data were expressed as Mean and standard deviation

**Table 3:** Levels of D-dimer for patients who underwent TKA with or without tourniquet use

D-dimer	Tourniquet (µg/l)	Non-tourniquet (µg/l)	*P* value
Preoperation	203.48 ± 117.54	334.64 ± 445.23	0.16 (t-test)
Postoperative day 1	2709.72 ± 1592.11	2862.88 ± 1521.42	0.73 (t-test)
Postoperative day 3	1631.64 ± 962.68	1849.60 ± 1742.07	0.59 (t-test)
Postoperative day 7	1920.48 ± 834.22	2653.72 ± 1378.58	**0.03 (t-test)**
Postoperative day 14	1361.76 ± 424.16	1985.52 ± 1531.07	0.06 (t-test)

µg/l, microgram. Measurement data were expressed as Mean and standard deviation.

### Postoperative Complications

Although the tourniquet group had almost twice as many complications as the nontourniquet group with regard to thromboembolic and nonthromboembolic events and overall complications, this difference was not statistically significant (P > 0.05) (Table 4).

Considering nonthromboembolic complications (Table 5), local complications in the tourniquet group were significantly higher than those in the nontourniquet group (P = 0.03). The most common local complications in the tourniquet group were bruising (2 cases), blisters (1 case), pain (2 cases), and superficial infections (1 case).

**Table 4:** Postoperative complications for patients who underwent TKA with or without tourniquet use

	Tourniquet	Non-tourniquet	*P* value
Thromboembolic events	2/25	1/25	1.00 (Chi-square test)
Non-thromboembolic events	7/25	3/25	0.09 (Chi-square test)
Overall complications	9/25	4/25	0.06 (Chi-square test)

**Table 5:** Postoperative non-thromboembolic complications for patients who underwent TKA with or without tourniquet use

	Tourniquet	Non-tourniquet	*P* value
Local complications	6/25	0/25	**0.03 (Chi-square test)**
Gastrointestinal complications	1/25	0/25	1.00 (Chi-square test)
Anterior knee pain	2/25	3/25	1.00 (Chi-square test)
Urinary tract infection	0/25	1/25	1.00 (Chi-square test)

## Discussion

The results of this study showed that patients in both the tourniquet and nontourniquet groups had the lowest postoperative hemoglobin levels from postoperative Days 3 to 7. In 47 patients, the lowest hemoglobin level was on postoperative Day 3 or 7, accounting for 94% of all patients. Postoperative hemorrhage gradually improves at 72 h after TKA, and patients often have the lowest hemoglobin level from postoperative Days 3 to 7. Thus, surgeons should pay attention during this period to ensure perioperative safety, especially for patients with mild anemia before surgery. Our study showed that tourniquet use in TKA had no significant effect on blood loss or the blood transfusion rate compared with nontourniquet use. A possible explanation is that hemostasis must be more meticulous and effective when a tourniquet is not used [11]; another possibility is that tourniquet-induced ischemia and reperfusion and secondary hyperfibrinolysis increase the concealed hemorrhage volume [12].

We typically use tourniquets during surgery. Tourniquet use reduces intraoperative hemorrhage and provides a clear surgical advantage for TKA; thus, it should theoretically aid surgeons in reducing the length of the operation [13]. However, our study found that tourniquet use does not affect the duration of surgery. Similar studies have also confirmed that tourniquet use has no significant correlation with the length of surgery in TKA [11, 14]. Because of the use of interrupted sutures, the length of our surgery is slightly longer than that of other researchers. Based on our own surgical experience, decreased bleeding in the surgical field can be achieved by using electrocoagulation hemostasis of the superior medial, inferior medial, and inferior lateral genicular arteries, followed by appropriate blood pressure control. All patients enrolled in our study underwent primary TKA, without any severe preoperative deformity; thus, complex osteotomy and soft tissue balancing were unnecessary, and the surgical procedures were relatively uncomplicated. In addition, the procedures limb exsanguination and tourniquet inflation and deflation (we usually inflate the tourniquet before cutting the skin and deflate it after the suture is complete) may increase the length of surgery. These reasons account for the absence of differences in the length of surgery between the tourniquet and nontourniquet groups.

The postoperative complications of TKA can be classified as thrombotic and nonthrombotic events. Thrombotic events include DVT and pulmonary embolism, whereas other complications are classified as nonthrombotic events. The results of our study showed that the overall complications and thrombotic and nonthrombotic events in the two groups were not significantly different. However, the tourniquet group had a significantly higher rate of local complications at the site of the affected limb, such as thigh bruising, blisters, pain, fat liquefaction, and superficial infections. Despite no significant difference, the incidence of local complications in the tourniquet group (36%), which was twice as high as that in the nontourniquet group (16%), should be taken into consideration. Direct tourniquet pressure on the thigh can cause local complications. Wound complications may be caused by hypoxic conditions due to tourniquet use, resulting from insufficient oxygen supply for wound healing and angiogenesis, as well as decreased macrophage and fibroblast migration [15, 16]. In addition, reactive hyperemia following tissue ischemia leads to lower extremity swelling that increases wound tension and further reduces the oxygen supply around the wound, thereby affecting wound healing [10]. A continuous wound exudate also increases the infection rate [17]. In contrast, not using a tourniquet may lead to a poorer surgical field of view, greater intraoperative bleeding, destroy more biology with more coagulation of bleeders, ultimately affecting the patient’s recovery process.

In this study, the incidence rate of thrombotic events was 6%, which was lower than that of 40% reported for the Asian population [18]. This decrease in incidence rate might be due to effective prevention of thrombosis after surgery, as well as our sample size and DVT diagnostic methods. No difference was found in the incidence rate of thrombotic events between the tourniquet and nontourniquet groups. The D-dimer level is often used as a diagnostic or exclusion indicator to predict the incidence of DVT. In this study, postoperative plasma D-dimer levels increased significantly and peaked on postoperative Days 1 and 7. No differences were observed in D-dimer levels on postoperative Days 1, 3, and 14 between the two groups, except on postoperative Day 7, when the D-dimer level in the tourniquet group was lower than that in the nontourniquet group. This result is consistent with the absence of a difference in the incidence of thrombotic events between the two groups. Many studies have shown that tourniquet use can enhance fibrinolysis, which leads to elevated D-dimer levels [19]. For the nontourniquet group, the anesthesiologist administered a large volume of crystalloids and colloids during the surgery to maintain the patient’s blood pressure, resulting in peripheral vasoconstriction, which promotes thrombosis [12]. For this reason, it may counteract the significant difference in D-dimer levels between the tourniquet and nontourniquet groups on postoperative Days 1 and 3. The following are possible reasons for the peaking D-dimer level on postoperative Day 7 and the higher level in the nontourniquet group than in the tourniquet group [20]: (1) two peaks of postoperative D-dimer levels occurred on postoperative Days 1 and 7, respectively, and (2) the second peak on postoperative Day 7, as confirmed by Shiota et al., is associated with reactivation of fibrinolysis. Tourniquet use in the tourniquet group promoted earlier fibrinolysis, which may have peaked before postoperative Day 7 when compared with the nontourniquet group, as opposed to delaying fibrinolysis in the nontourniquet group. Hence, the nontourniquet group had a higher D-dimer level on postoperative Day 7 than the tourniquet group.

Whether nontourniquet use affects prosthesis fixation or causes loosening of the prosthesis is the most concerning issue. No current evidence exists indicating that surgery without tourniquet use affects the long-term survival of the prosthesis [13]. Medical practices have demonstrated that bone cement achieves satisfactory results in hip arthroplasty, for which we cannot use a tourniquet to control bleeding of the milled acetabular surface. In the process of fixing the knee with bone cement, lowering the blood pressure and pulse lavage enable effective penetration of the cement into the bones, firmly fixing the prosthesis.

The clinical relevance of this study lies in the fact that although the use of tourniquets in TKA is still a controversial topic and there is no standard conclusion, the results of this study provide a reference for joint surgery clinician choice. Therefore, joint surgeons can choose to use tourniquets according to their own preferences, but when using tourniquets, certain measures need to be taken to reduce the occurrence of local complications.

The cost-effectiveness analysis of this study mainly focuses on the length of hospital stay and its medical expenses. The two groups of patients had similar outcomes in terms of blood loss, operation time and drainage volume. However, the incidence of local complications in the nontourniquet group was significantly lower than that in the tourniquet group. Local complications caused by the use of tourniquets may prolong the patient’s hospitalization time and increase corresponding medical expenses. In addition, as tourniquets require additional medical equipment (tourniquets and inflatable pumps), from this perspective, the cost-effectiveness ratio of not using tourniquets is better.

The main advantage of this study is that it was a randomized controlled study, and the difference between the two treatment group interventions (using or not using tourniquets) is simple and clear.

Our study also has limitations. This was a single-center randomized controlled study, the number of cases was limited, and our follow-up time was short, with possible errors in the statistical results. We will carry out multicenter large sample size research in future work to improve the quality of our research.

## Conclusion

Tourniquet use in TKA does not reduce the operation time, overall blood loss, or postoperative blood transfusion rate. Although tourniquet use in TKA has no significant effect on the incidence of postoperative overall complications, the incidence of postoperative local complications increases with the application of a tourniquet. Therefore, joint surgeons can choose to use or not use tourniquets according to their own preferences; if using tourniquets, they need to take certain measures to reduce the occurrence of local complications.

## Data Availability

The datasets during and/or analysed during the current study available from the corresponding author on reasonable request.

## Abbreviations

TKA: Total knee arthroplasty
OA: Steoarthritis
DVT: Deep venous thrombosis
BMI: Body mass index
IRB: Institutional review board
CBL: The calculated total blood loss
TBL: Total blood loss
SDs: Standard deviations
ANOVA: Repeated measurement analysis of variance
